# Achievement at school and socioeconomic background—an educational perspective

**DOI:** 10.1038/s41539-018-0022-0

**Published:** 2018-03-23

**Authors:** Sue Thomson

**Affiliations:** Australian Council for Educational Research, Camberwell, VIC Australia

## INTRODUCTION

Educational achievement, and its relationship with socioeconomic background, is one of the enduring issues in educational research. The influential Coleman Report^1^ concluded that schools themselves did little to affect a student’s academic outcomes over and above what the students themselves brought to them to school—‘the inequalities imposed on children by their home, neighbourhood and peer environment are carried along to become the inequalities with which they confront adult life at the end of school’ (p. 325). Over the intervening 50 years, much has been added to the research literature on this topic, including several high-quality meta-analyses. It has become ubiquitous in research studies to use a student’s socioeconomic background, and that of the school they attend, as contextual variables when seeking to investigate potential influences on achievement.

The two articles in this issue of Science of Learning touch on aspects of this discussion rarely included in the educational research literature. The article by Smith–Wooley et al.^2^ asks whether whether it is the influence of the student socioeconomic background that is the greater influence or whether the parents are passing down intellectually advantageous genes to their offspring. In contrast, the article by van Dongen et al.^3^ suggests that that it is likely a combination of genetics and socioeconomic background, and they examine the effect of environment on the epigenetic status of genes that are involved in learning and memory.

### What do we mean by socioeconomic background?

The definition of socioeconomic background used varies widely, even across educational research. In the Organisation for Economic Cooperation and Development’s (OECD) rigorous large-scale international assessment of more than 70 countries over 15 years, the Programme for International Student Assessment (PISA), socioeconomic background is represented by the index of Economic, Social and Cultural Status, which is a composite score derived by principal components analysis and is comprised of the International Socioeconomic Index of Occupational Status; the highest level of education of the student’s parents, converted into years of schooling; the PISA index of family wealth; the PISA index of home educational resources; and the PISA index of possessions related to 'classical' culture in the family home.^4^

However, examining Sirin’s^5^ meta-analysis of the research into socioeconomic status and academic achievement finds that many studies use a combination of one or more of parental education, occupation and income, others include parental expectations, and many simply use whether the student gets a free or reduced-price lunch. The latter factor is most commonly used as it is readily available from school records rather than having to ask questions about occupation and education of students or parents, yet Hauser^6^ as well as Sirin have argued that it is conceptually problematic and should not be used. Other studies have used family structure,^7,8^ family size,^9^ and even residential mobility.^10^

Sirin’s meta-analysis, however, found that the traditional definitions of socioeconomic background were not as strongly related to educational outcomes for students from different ethnic backgrounds, for those from rural areas, or for migrants. Its use in developing countries is particularly problematic. For example, in examining the effect of household wealth on educational achievement, Filmer & Pritchett^11^ found that many poor children in developing countries either never enrol in school or attend to the end of first grade only. Even within developing countries, the gap in enrolment and achievement between rich and poor was found to be only a year or two, in other countries 9 or 10 years. Often in developing countries low achievement and enrolment is attributable to the physical unavailability of schools.

Similarly education achievement is measured in many ways—achievement on a set test in certain subject areas, completion of numbers of years of schooling, entrance to university, for example.

What does this mean for educators when they are reviewing the research? It means that they need to exercise some caution. The results and the conclusions will obviously vary, as the research is, essentially, looking at different influencers and not the same influence each time. So, when the argument is made that the relationship is not stable, this may well be because the variable under consideration is different.

### School-level socioeconomic background

While the Coleman Report concluded that schools themselves added little to effect outcomes, the school environment, in particular the social background of a student’s peers at the school, has certainly been found to be positively related to student achievement. On average, a student who attends a school in which the average socioeconomic status is high enjoys better educational outcomes compared to a student attending a school with a lower average peer socioeconomic level.^12,13^

### Relationship between achievement and student socioeconomic background

There is some discussion about the size of the effect, however the relationship between a student’s socioeconomic background and their educational achievement seems enduring and substantial. Using data from PISA, the OECD have concluded that 'while many disadvantaged students succeed at school … socioeconomic status is associated with significant differences in performance in most countries and economies that participate in PISA. Advantaged students tend to outscore their disadvantaged peers by large margins' (p. 214).^14^ The strength of the relationship varies from very strong to moderate across participating countries, but the relationship does exist in each country. In Australia, students from the highest quartile of socioeconomic background perform, on average, at a level about 3 years higher than their counterparts from the lowest quartile.^15^ Over the 15 years of PISA data currently available, the size of this relationship, on average, has changed little, and over the now 50 years since the publication of the Coleman Report, the gap between advantaged and disadvantaged students remains.

### How are these effects transmitted?

What the continued gap between advantaged and disadvantaged students highlights is that despite all the research, it is still unclear how socioeconomic background influences student attainment.

There are those that argue that the relationships between socioeconomic background and educational achievement are only moderate and the effects of SES are quite small when taking into account cognitive ability or prior achievement.^16^ Cognitive ability is deemed to be a genetic quality and its effects only influenced to a small degree by schools. Much of the body of research, particularly that generated from large-scale international studies, would seem to contradict this reasoning.

Others have argued that students from low socioeconomic level homes are at a disadvantage in schools because they lack an academic home environment, which influences their academic success at school. In particular, books in the home has been found over many years in many of the large-scale international studies, to be one of the most influential factors in student achievement.^15^ From the beginning, parents with higher socioeconomic status are able to provide their children with the financial support and home resources for individual learning. As they are likely to have higher levels of education, they are also more likely to provide a more stimulating home environment to promote cognitive development. Parents from higher socioeconomic backgrounds may also provide higher levels of psychological support for their children through environments that encourage the development of skills necessary for success at school.^17^

The issue of how school-level socioeconomic background effects achievement is also of interest. Clearly one way is in lower levels of physical and educational resourcing, but other less obvious ways include lower expectations of teachers and parents, and lower levels of student self-efficacy, enjoyment and other non-cognitive outcomes.^15^ There is also some evidence that opportunity to learn (particularly in mathematics) is more restricted for lower socioeconomic students, with ‘systematically weaker content offered to lower-income students [so that] rather than ameliorating educational inequalities, schools were exacerbating them’.^18^

## Conclusions

If the role of education is not simply to reproduce inequalities in society then we need to understand what the role of socioeconomic background more clearly. While much research has been undertaken in the past 50 years, and we are fairly certain that socioeconomic background does have an effect on educational achievement, we are no closer to understanding how this effect is transmitted. Until we are, it will remain difficult to address. In this edition of Science of Learning, two further contributions to this body of knowledge have been added—and perhaps indicate new paths that need to be followed to develop this understanding.
